# LIGYSIS-web: a resource for the analysis of protein-ligand binding sites

**DOI:** 10.1093/nar/gkaf411

**Published:** 2025-05-16

**Authors:** Javier S Utgés, Stuart A MacGowan, Geoffrey J Barton

**Affiliations:** Division of Computational Biology, School of Life Sciences, University of Dundee, Dow Street, Dundee DD1 5EH Scotland, UK; Division of Computational Biology, School of Life Sciences, University of Dundee, Dow Street, Dundee DD1 5EH Scotland, UK; Division of Computational Biology, School of Life Sciences, University of Dundee, Dow Street, Dundee DD1 5EH Scotland, UK

## Abstract

LIGYSIS-web is a free website accessible to all users without any login requirement for the analysis of protein-ligand binding sites. LIGYSIS-web hosts a database of 65,000 protein-ligand binding sites across 25,000 proteins. LIGYSIS sites are defined by aggregating unique relevant protein–ligand interfaces across different biological assemblies of the same protein deposited on the PDBe. Additionally, users can upload their own structures in PDB or mmCIF format for analysis and subsequent visualisation and download. Ligand sites are characterised using evolutionary divergence from a multiple sequence alignment, human missense genetic variation from gnomAD and relative solvent accessibility to obtain accessibility-based cluster labels and scores indicating likelihood of function. These results are displayed in the LIGYSIS web server, a Python Flask web application with a JavaScript frontend employing Jinja and jQuery to link the 3Dmol.js structure viewer with dynamic tables and Chart.js graphs in an interactive manner. LIGYSIS-web is available at https://www.compbio.dundee.ac.uk/ligysis/, whilst the source code for the analysis pipelines and web application can be accessed at https://github.com/bartongroup/LIGYSIS, https://github.com/bartongroup/LIGYSIS-custom and https://github.com/bartongroup/LIGYSIS-web, respectively.

## Graphical Abstract

**Graphical Abstract:**
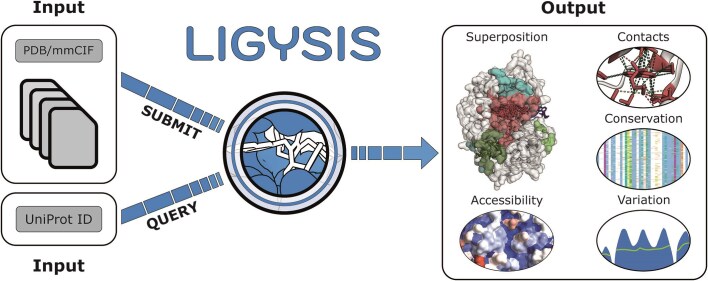

## Introduction

Ligands play a critical role in protein function acting as natural co-factors, substrates, inhibitors and drugs in disease therapy. Identifying the protein regions where these molecules bind, understanding the mode in which they do so and characterising that interface is therefore key to understanding and modulating protein function. The UniProt knowledgebase (UPKB) catalogues 248 million protein sequences [1–3]. While structure models for most of these proteins are available through resources such as the AlphaFold Database [4, 5] and other providers [6–8], only a small fraction present residue-level functional annotations in UniProt – 55,000 (0.02% of UPKB) – or include biologically relevant ligands co-crystallised in the Protein Data Bank Europe (PDBe) [9–11] – 29,000 (0.01%). The significant expense and time required for experimental validation underscores an urgent need for computational methods to characterise ligand sites systematically and highlight residues likely to be relevant to protein function.

Previously, Utgés *et al.* [12] proposed a novel method for defining ligand binding sites from multiple protein–ligand complexes derived from fragment screening, a widely used technique for identifying hit molecules in drug discovery [13–16]. Unlike traditional approaches, which rely on spatial ligand positioning [17–20], Utgés *et al.* use protein–ligand interactions to group ligands into binding sites. Sites were categorised by solvent accessibility into four clusters, each showing differential enrichment in known functional sites. These clusters provide a basis for ranking sites by likelihood of functional significance. This method was subsequently applied to the entire PDBe, resulting in the *LIGYSIS* dataset, which Utgés and Barton [21] employed to benchmark protein–ligand binding site prediction methods. Unlike databases such as PDBbind [22–24], BindingDB [25–27], or binding MOAD [28–30], which focus on the binding affinity of single protein–ligand complexes, LIGYSIS defines binding sites by the aggregation of *all* available relevant protein–ligand complexes for a protein. LIGYSIS sites thus identify all amino acids observed in binding for each part of the protein.

In this work, we introduce LIGYSIS-*web*, a resource for the analysis of protein–ligand binding sites. LIGYSIS-web hosts the LIGYSIS database, comprising 64,782 ligand binding sites, defined from 435,038 biologically relevant ligands, across 25,003 proteins with protein–ligand complexes deposited in the PDBe. Furthermore, users can submit their own structures to the LIGYSIS web server for analysis, visualise the results in a dynamic and interactive manner, and download them for further analysis.

## LIGYSIS pipeline

LIGYSIS is a pipeline for the analysis of protein–ligand binding sites built on the Protein Data Bank Europe Knowledge Base (PDBe-KB) [31, 32] and PDBe GRAPH and REST application programming interfaces (API) [33]. There are ≈65,000 proteins with at least one experimentally determined structure deposited in the PDBe [11]. ≈29,000 of these (45%) present at least one structure in complex with a biologically relevant ligand as defined by BioLiP [34]. A structural segment is defined by the PDBe-KB as a protein region with structural coverage that maps to a contiguous section of their corresponding UniProt sequence [35]. A protein can have multiple segments. For example, each domain of a multi-domain protein for which there are independent structures would correspond to a segment.

### Ligand binding site definition

The ligand binding site definition and characterisation approaches described here build on our previous work on fragment screening [12]. Ligand sites were defined by grouping biologically relevant protein–ligand interfaces across PISA-defined [36] biological assemblies of the multiple structures mapping to a UniProt accession. Assemblies were downloaded from PDBe via ProIntVar [37] and inter-atomic contacts calculated with pdbe-arpeggio [38]. Note that pdbe-arpeggio currently does not support multiple occupancy atoms, and so ligands with atom occupancy ≠ 1 were not considered in our analysis. This affects 6256 structures (6% of LIGYSIS dataset). Refer to [21] for full details on this approach. Around 64,782 sites were defined from 435,038 ligands across 26,260 structural segments. These segments mapped to 25,003 different proteins, which were represented by 104,456 structures.

### Alignments and variants

The canonical sequence for each UniProt accession was used to perform a homologue sequence search in SwissProt [39]. jackHMMER [40] was employed with three iterations to generate a multiple sequence alignment (MSA). Amino acid divergence was quantified with the normalised [41] divergence score (DS) *N_Shenkin_* [42]. Genetic missense variants mapping to human sequences in the MSA were retrieved from gnomAD [43] using VarAlign [44]. Missense enrichment scores (MES), i.e. odds ratio (OR), were calculated for alignment columns [45] and 95% confidence intervals and *p*-values used to evaluate their significance [46]. MES could not be calculated for proteins with no human homologues in the alignment.

### RSA-based clustering and score

Accessible surface area was calculated by DSSP [47] via ProIntVar [37] and normalised [48] to relative solvent accessibility (RSA). A Keras [49] multilayer perceptron (MLP) [50] developed in our previous work [12] was employed to predict site RSA-based cluster labels: C1, C2, C3 and C4. These clusters are differentially enriched in functional sites annotated in UniProt [51] (OR_C1_ ≈ 28 × OR_C4_). For a binding site $i$, a functional score $F{{S}_i}$ is calculated with Equation 1 as the dot product of the ${{P}_i}$ and $F$ vectors. In this equation, ${{p}_{{{i}_j}}}$ represents the probability of site $i$ belonging in Cluster $j$, and ${{f}_j}$ denotes the proportion of annotated functional sites within Cluster $j$. The probabilities ${{p}_{{{i}_j}}}$ are derived from the vector $P$ (Equation 2), which is returned by the MLP and provides the probabilities of a site belonging to each class. The vector *F* (Equation 3) contains the proportions of functional sites in each cluster, which were determined through hierarchical clustering and functional classification of the human subset of the LIGYSIS dataset, composed of 13,000 sites across 3500 proteins. Both the functional label and score serve as metrics that indicate the likelihood of a binding site being functional and can be used to rank binding sites within a protein.


(1) \begin{eqnarray*}
F{{S}_i} = {{P}_i} \cdot F = \ \mathop \sum \limits_{j = 1}^4 {{p}_{{{i}_j}}}{{f}_j}
\end{eqnarray*}

(2) \begin{eqnarray*}
{{P}_i} = \left[ {{{p}_{{{i}_1}}},\ {{p}_{{{i}_2}}},\ {{p}_{{{i}_3}}},\ {{p}_{{{i}_4}}}} \right]
\end{eqnarray*}

(3) \begin{eqnarray*}
F = \left[ {{{f}_1},\ {{f}_2},\ {{f}_3},\ {{f}_4}} \right] = \left[ {0.52,\ 0.18,\ 0.05,\ 0.04} \right]
\end{eqnarray*}

## LIGYSIS customised pipeline

The LIGYSIS *customised* pipeline is used for the analysis of user-submitted structures to the server. The customised pipeline neither relies on the PDBe-KB for mapping chains to a protein nor for transformation matrices. Instead, STAMP [52] is used to superpose the uploaded structures, which can be in PDB (*.ent*, *.pdb*) or mmCIF (*.cif*) format. However, all structures in the set must be in the same format, e.g. either all PDB or all mmCIF. Structures must present the same number of protein chains, either all monomers, dimers, trimers, etc. The LIGYSIS web server currently supports only homomeric protein–ligand complexes, i.e. complexes between *N* copies of a single protein sequence and any number of ligands. For structures mapping to a protein in UniProt, it is required to submit the corresponding UniProt ID, so residues across different structures can all be mapped to the same reference. If the submitted structures represent a protein not in UniProt, this field can be left blank. The residue numbering employed will be the structure one, so structures are expected to present the same numbering scheme.

## Server architecture

The LIGYSIS web server is implemented using the Python Flask framework [53], with dynamic content rendered via Jinja templates [54] and data transferred through Flask routes. The frontend is structured using HTML for content and layout and a combination of Bootstrap [55] and plain CSS [56] for styling. JavaScript [57] enhances interactivity by integrating a 3Dmol.js structure viewer [58, 59] for molecular visualisation, interactive tables and Chart.js graphs [60]. Asynchronous updates are facilitated by jQuery [61] and AJAX [62] requests, enabling dynamic data exchange between the client and server. The LIGYSIS web server can be accessed from: https://www.compbio.dundee.ac.uk/ligysis/.

User job submission is handled through Slivka-bio v0.8.3 [37, 63, 64] and jobs run on the School of Life Sciences, University of Dundee HPC infrastructure. Once the job execution has finished, result files are served to the client and displayed in the same way as the LIGYSIS database entries.

## LIGYSIS web server

### Results page

The LIGYSIS web server can be used in two modes: to explore the LIGYSIS dataset or to submit a set of structures for analysis to the LIGYSIS pipeline. The LIGYSIS dataset comprises 25,003 proteins with deposited structures of proteins bound to BioLiP biologically relevant ligands on the PDBe. Results can be explored by searching for a UniProt accession, entry, or name of the protein of interest. Results for user-submitted jobs can be accessed through the provided link when submitting the job, or through the summary table at the user session tab.

**Figure 1.**
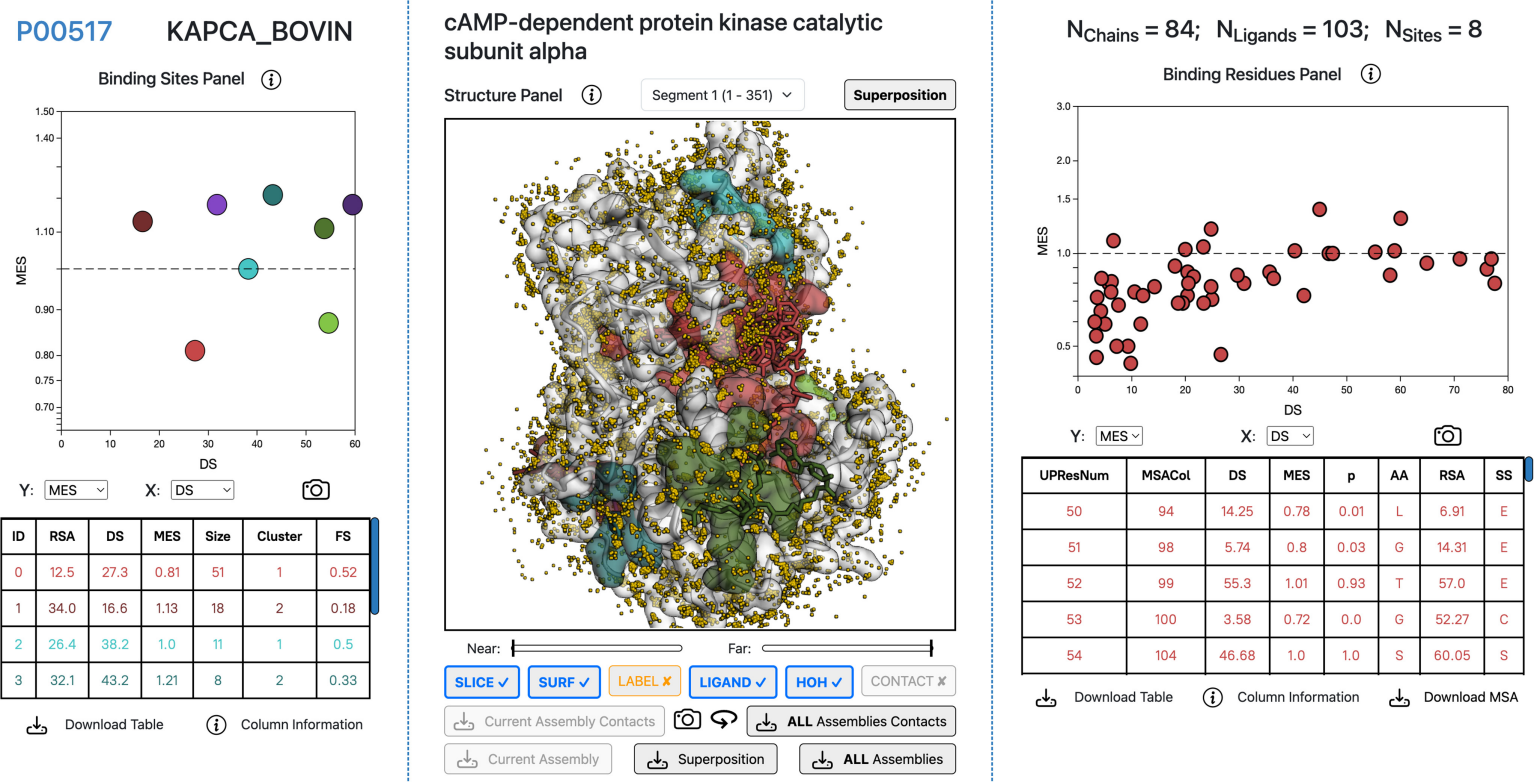
LIGYSIS-web results page. The results page is divided into three panels: Binding Sites (left), Structure (centre), and Binding Residues (right) panels. In this example, 103 ligands across 84 structures have been clustered into eight different ligand binding sites for bovine cAMP-dependent protein kinase catalytic subunit alpha, PKA C-alpha (P00517). This is the only segment of PKA C-alpha and covers its whole sequence (1-351). Chart.js scatter points and table rows represent binding sites and their average features on the Binding Sites Panel, whereas they represent individual amino acid residues on the Binding Residues Panel. Both panels interact with the 3Dmol.js central structure viewer through hover and click events. Below this viewer, function buttons can be found to hide/show slab controls, surfaces, labels, ligands, water molecules and protein-ligand contacts. Structures and contact data can also be downloaded from these buttons. Surfaces, ligands and water molecules are displayed in this screenshot.

Fig. 1 illustrates the LIGYSIS-web main results page. This page is divided into three panels, from left to right: Binding Sites, Structure, and Binding Residues panels. The results page is the same when exploring a LIGYSIS entry and user job results. At the top of the panels, general information about the target protein can be found, from left to right: UniProt accession identifier (link to UniProt), entry, protein names, and number of chains, ligands, and defined binding sites for the target protein.

### Binding sites panel

**Figure 2.**
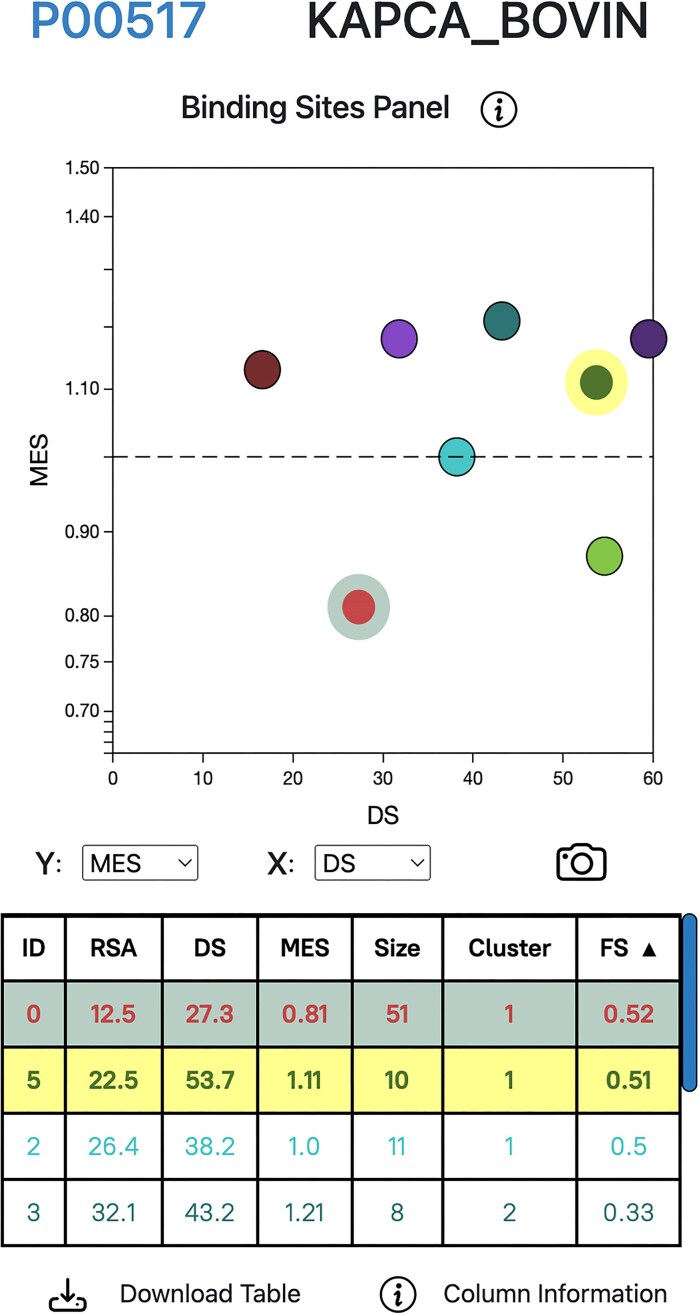
LIGYSIS-web results page Binding Sites panel. UniProt accession identifier and entry name for cAMP-dependent protein kinase catalytic subunit alpha (P00517) at the top. Below, Chart.js scatter of MES (log_10_ scale) versus DS for the eight binding sites defined for PKA C-alpha. The dashed line indicates neutrality, i.e. missense variation within the site is no different than at the rest of the protein. Binding Site 2 (over the dashed line) is an example of this. In contrast, Binding Site 0 (highlighted below dashed line), the known active site of the kinase, is depleted in missense variation (MES = 0.81). Additionally, it is buried (RSA = 12%), conserved across homologues (DS = 27/100) and presents a high functional score (FS = 0.52). Binding sites with low DS and MES (lower-left quadrant) are most likely to be functional, whereas those at the top-right are least likely to have biological effect when bound a ligand, e.g. BS6 (rightmost point). The Binding sites table is sorted by FS. Binding Site 0 (clicked) and Binding Site 5 (hovered over) are highlighted.

Fig. 2 depicts the Binding Sites panel of the results page. This panel is formed by a dynamic Chart.js canvas and a table. Both are displaying the mean binding site features, calculated from averaging the features of the residues forming the site. These features are RSA, normalised Shenkin DS, MES, the size of the site, i.e. number of amino acid residues, the RSA-derived cluster label and its associated functional score (FS). The table rows can be sorted by any of these variables and the axes of the chart dynamically changed. MES uses log_10_ scale, as it is an OR. The chart as well as the table are linked to the structure viewer by hover and click events. Hovering over a data point/row will temporarily display the side chains of the site residues, whereas clicking on it would fix them in the view until another site is clicked or the current one unclicked. Refer to Fig. 2 legend for more details on how to interpret this graph.

### Structure panel

**Figure 3.**
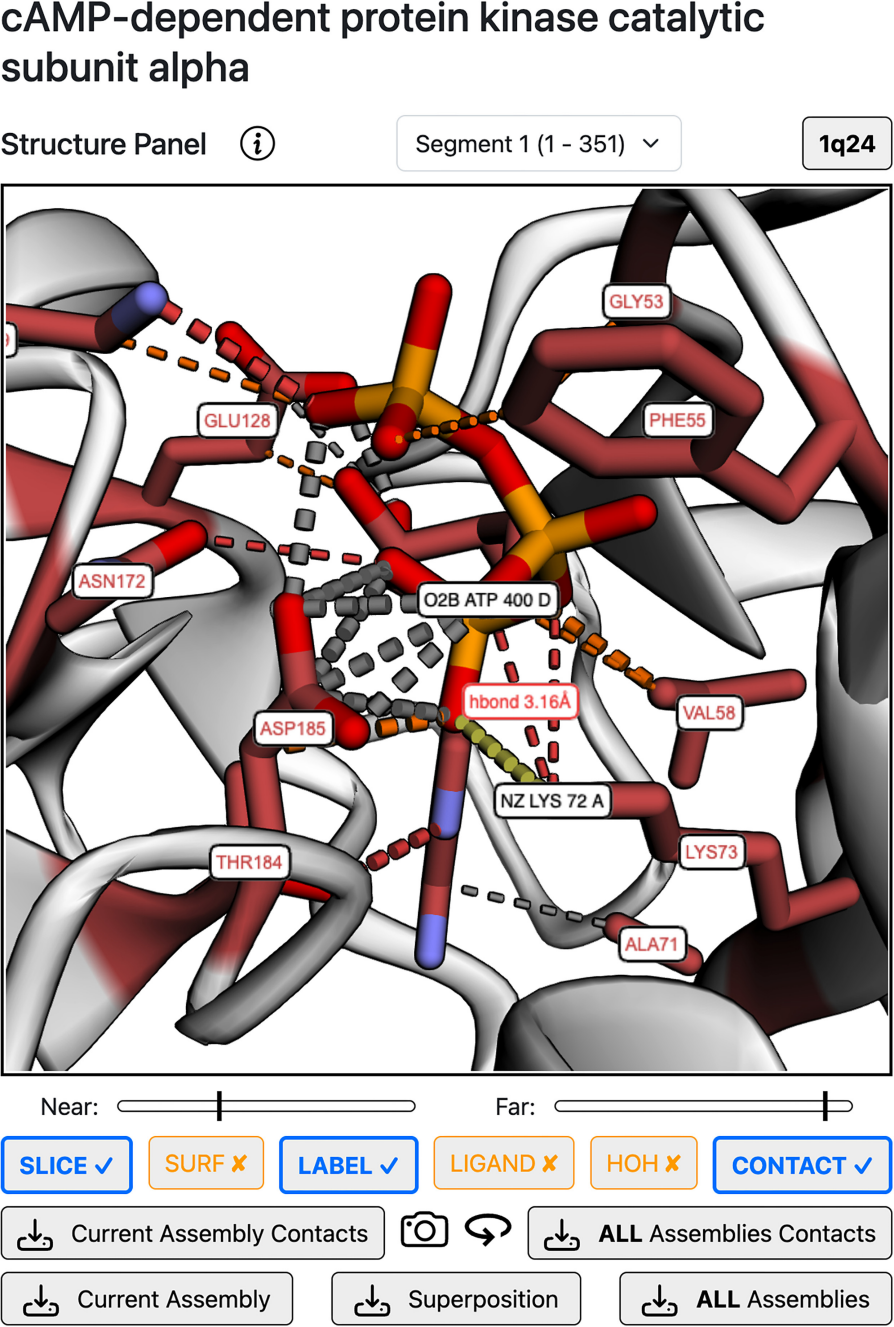
LIGYSIS-web results page Structure Panel. Preferred biological assembly of PKA C-alpha (P00517) PDB: 1Q24 [82] with displayed interactions with adenosine tri-phosphate (ATP). Water molecules and ligand-binding residue labels are displayed. Interaction label between NZ atom of LYS73 chain: A and O2B of ATP 400 chain: D is displayed on hover. This is a hydrogen bond and the distance between the atoms is of 3.16 Å. Residues in contact with multiple ligand atoms, as LYS73, which forms hydrogen bond and ionic interactions with three atoms, or ASP185, which interacts with four ATP atoms, are likely to be more relevant for the binding mode than residues with a single contribution to the binding interface, as ALA71.

Fig. 3 depicts the central Structure panel of the results page. This panel is divided in three parts: the main 3Dmol.js viewer, in the centre, segment, and structure selectors above it, and the structure buttons below it. The Segment selector is a drop-down menu showing the segment identifier and its protein sequence coordinates. The Structure selector is a drop-up menu that allows the user to swap between the Superposition view (default) and individual biological assemblies. The Superposition view consists of a white cartoon representation of a segment representative chain and the superposed ligands coloured by binding site. Superposed ligands are shown by default and can be hidden by clicking on the *“LIGAND”* button and displayed if clicked again. The *“SURF”* button will display the protein chain surface, which will be white for non-ligand binding residues and coloured for binding residues. The same applies to water molecules and the “*HOH*” button. To see the labels of a clicked binding site, click on the “*LABEL*” button, and click again to hide them. The “*CONTACT*” button is disabled for the superposition view but can be clicked when exploring an assembly or structure. The relevant ligands, as well as the residues interacting with them will be coloured based on their site, and dashed cylinders depicting the protein–ligand interactions calculated by pdbe-arpeggio will be displayed. They are coloured based on the Arpeggio colour scheme, e.g. green for hydrophobic, red for polar, or yellow for ionic [38]. The width of these cylinders is representative of the distance between the atoms. Thicker cylinders denote a clash between the Van der Waals (VDW) radii of the atoms (closer) whilst thinner cylinders indicate interaction between the VDW radii of the atoms. The view can also be sliced between two planes to focus on a region of interest. The slab or slice controls are displayed with the “*SLICE*” button. Hiding them does not reset the slab, just hides the controls. Clicking on the “*SLICE*” button again will show the controls and allow for further slab adjustment. Clicking on the circular arrow generates a spin animation on the *Y-*axis. Clicking on any ligand atom on the structure viewer links to the new PDBe-KB ligand pages [65], providing enhanced ligand annotations and a holistic view of small molecules for their biological context.

### Binding residues panel

**Figure 4.**
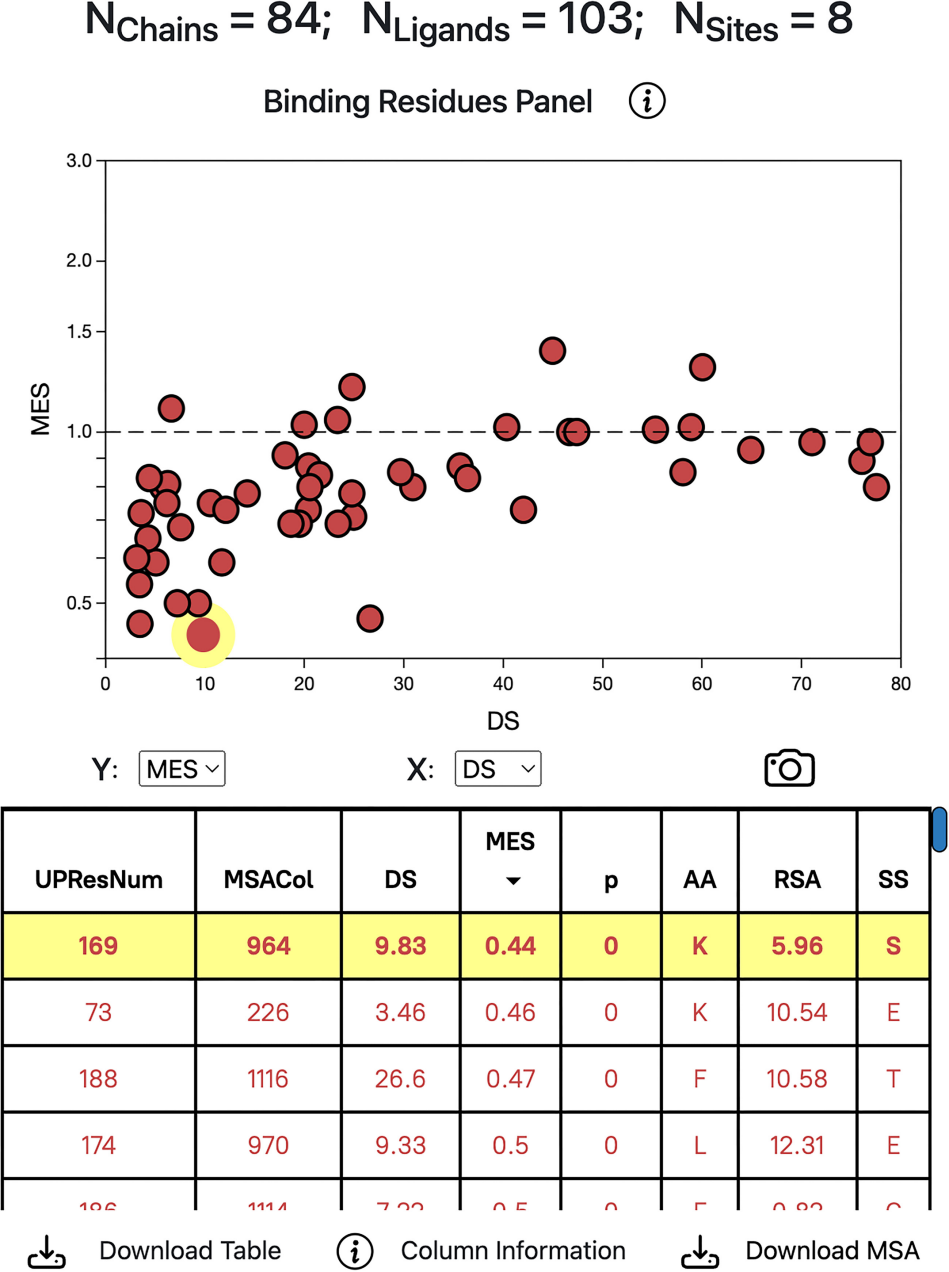
LIGYSIS-web results page Binding Residues Panel. MES vs normalised Shenkin DS for the 51 residues of Binding Site 0 of PKA C-alpha. This site corresponds to the ATP binding site of PKA C-alpha. Most residues on this site present MES < 1 (below the dashed line), indicating depletion in human missense variation resulting from selective pressure or constraint. Missense-depleted residues that are also conserved are most likely to be functional and cause an effect on the protein function if targeted. An example is LYS169, which is buried (RSA = 6%), conserved across homologues (DS = 10) and depleted in missense variation (MES = 0.44, *p* ≈ 0). This residue is known to be functionally relevant as it interacts with ATP, which agrees with the results displayed on the table. Residues are sorted by MES on the table (lowest at the top). LYS169 is the most depleted and is hovered over (highlight).

Fig. 4 illustrates the Binding Residues Panel, which is similar to the Binding Sites Panel and includes a chart and a table. However, data points and rows correspond to individual residues, and not the site as a whole, as they do in the Binding Sites Panel. The table displays the UniProt residue number (UPResNum), the column in the multiple sequence alignment (MSACol), the *N_Shenkin_* DS, MES, associated *p*-value, amino acid name (AA), RSA and secondary structure (SS). SS corresponds to the original 8-state DSSP classification: 3_10_ helix (G), α-helix (H), π-helix (I), β-sheet (E), β-bridge (B), helix turn (T), bend (S) and coil (C) [47]. Variables on the chart axes can also be changed, and table rows are sorted by column. Only hover events are supported for this panel, clicking chart points or table rows has no effect. Refer to Fig. 4 legend for more details on how to interpret this graph.

### Data export

A *“Download Table”* button can be found on the *Binding Site* and *Binding Residues* panels. Clicking on this button will download the binding site or binding site residues tables, respectively, in CSV (*.csv*) format. The MSA from which the DSs are derived can also be downloaded in Stockholm format (*.sto*) by clicking on the *“Download MSA”* button. Additionally, (pdbe-arpeggio) protein–ligand contacts can be downloaded in tabular format by clicking on the *“Download Current Assembly Contacts”* and *“Download **ALL** Assemblies Contacts”* for the assembly currently being explored, or all of them. The first option is not available when exploring the *Superposition* view, since contacts are calculated on individual assemblies, and the second one will download a zipped folder of CSV files.

**Figure 5.**
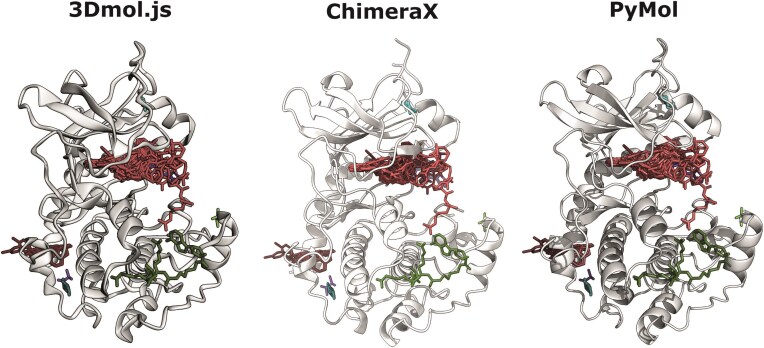
LIGYSIS-web supports ChimeraX and PyMol. LIGYSIS-web employs 3Dmol.js for visualisation. Additionally, it supports download of both superposition view as well as individual or all assemblies in the common structure viewers ChimeraX and PyMol. In this example, PDB: 1SVH, chain: A [83] is the representative chain for cAMP-dependent protein kinase catalytic subunit alpha (P00517), for which eight binding sites are defined from 103 ligands across 84 structures.

The ligand superposition view, i.e. representative chain with bound ligands across structures can be saved to ChimeraX (*.cxc*) or PyMOL (*.pml*) script files by clicking on *“Download Superposition”* and then selecting the preferred viewer. The same can be done for individual (*“Download Current Assembly”*) or all assemblies (*“Download **ALL** Assemblies”*). Fig. 5 exemplifies this with human PKA C-alpha (P00517). Screenshots of both graphs as well as 3Dmol.js view can be saved to PNG (*.png*) files by clicking on the camera icon on the corresponding panel.

## LIGYSIS-web analysis of bovine rhodopsin

G-protein coupled receptors (GPCR) comprise the largest protein receptor family in the human genome, with ≈800 members [66]. GPCRs have a regulatory role in most physiological processes including the visual, gustatory and smell sense, immune and nervous system activity, as well as in disease [67]. Consequently, GPCRs represent an important target in drug therapy, covering ≈35% of drugs approved by the Food and Drug Administration [68, 69]. GPCRs share a conserved seven-transmembrane helix fold connected by three intra- and three extra-cellular loops [70] and bind to a variety of endogenous ligands including peptides, ions, lipids, or neurotransmitters [71, 72]. Once activated by external stimuli, e.g. ligand binding, GPCRs employ G-proteins to interact with downstream effectors thus triggering intracellular signalling cascades [73]. To accommodate for the wide variety of substrates that GPCRs bind, specificity-determining positions (SDP) are required at the protein–ligand interface. These sites are unconserved across homologues and depleted in missense variation in human (UMD) [45].

**Figure 6.**
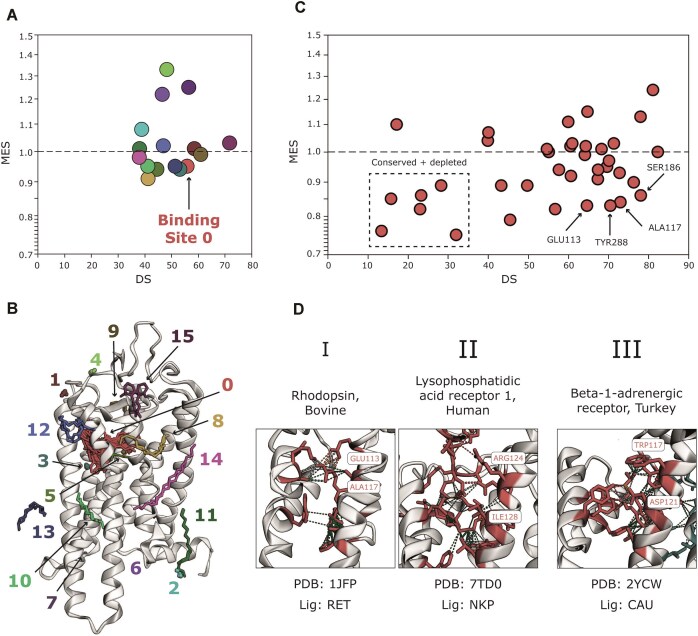
Analysis of bovine rhodopsin with LIGYSIS-web. (**A**) Scatter of average site MES vs DS for the 16 ligand binding sites defined for bovine rhodopsin (P06299). Binding Site 0 is divergent, or unconserved, (DS = 56) and depleted in missense variation (MES = 0.95); (**B**) Superposition view of 145 ligands of interest across 49 structures of rhodopsin visualised with 3Dmol.js; (**C**) MES versus DS for the 38 residues forming Binding Site 0. Residues within the dashed rectangle are conserved across homologues and depleted in missense variation in human, indicating common function and constraint across members of the family. However, GLU113, ALA117, SER186 and TYR288 in bovine rhodopsin are divergent within the family, yet constrained in human, suggesting a substrate specificity determining role for these residues; (**D**) Examples of multiple GPCRs binding to endogenous substrates (retinal and lysophosphatidic acid) and inverse agonist (carazolol). (**I**) Bovine rhodopsin binding to retinal (RET) – PDB: 1JFP [84]. (II) Human lysophosphatidic acid receptor 1 binding to oleoyl lysophosphatidic acid (NKP) – PDB: 7TD0 [85]. (III) Turkey beta-1-adrenergic receptor binding to carazolol (CAU) – PDB: 2YCW [86]. The same alignment position presents different amino acids across members of the family to accommodate for a variety of substrates. However, each of these residues is missense-depleted and forms ligand-specific interactions within each GPCR.

Fig. 6 illustrates how SDPs can easily be identified in LIGYSIS-web for the example of the bovine rhodopsin GPCR (P06299). Fig. 6A shows the average site missense enrichment vs divergence for the 16 binding sites defined in LIGYSIS from 145 ligands of interest across 45 structures (Fig. 6B). Binding Site 0 corresponds to the orthosteric GPCR site and is on average unconserved (DS > 50) and missense-depleted (MES < 1). Four of the 38 residues within this site are significantly depleted in missense variation (*p* < 0.05): GLU113, ALA117, SER186 and TYR288 (Fig. 6C). Fig. 6D exemplifies the specificity role of these positions by showing how different GPCRs bind to their substrates with different amino acids at these positions.

## Discussion

The LIGYSIS web server is a free open resource accessible to all users without any login requirement for the analysis of protein-ligand binding sites. It hosts the LIGYSIS dataset, an integrative protein-ligand complex dataset including 64,782 biologically relevant binding sites across 25,003 proteins with structures on the PDBe. Additionally, users can upload their structures for analysis, visualisation and download. LIGYSIS defines binding sites by clustering protein–ligand interactions and characterises them by evolutionary divergence, missense variation and solvent accessibility, thus offering insight into the likelihood of function of sites as well as individual residues. These results are interactively displayed on LIGYSIS-web, a Python Flask web application using Chart.js for dynamic graph rendering and 3Dmol.js for structure visualisation. The server can be accessed from this link: https://www.compbio.dundee.ac.uk/ligysis/.

Future steps might include implementing a search by ligand name or type, overcoming the current limitation of multi-occupancy ligand atoms, the implementation of heteromeric protein-ligand complexes or analysing predicted ligand binding sites by methods as P2Rank [74–78], fpocket [79, 80], or IF-SitePred [81].

## Data Availability

The code for the LIGYSIS-web Python Flask application can be found in our GitHub repository (https://github.com/bartongroup/LIGYSIS-web) and on Zenodo (https://zenodo.org/records/15309182). The code for the LIGYSIS pipeline, employed to generate the LIGYSIS dataset, which LIGYSIS-web explores can be found here: https://github.com/bartongroup/LIGYSIS and https://doi.org/10.5281/zenodo.14178252. The code for the LIGYSIS pipeline adapted to handle user-submitted jobs can be found in our repository: https://github.com/bartongroup/LIGYSIS-custom and https://zenodo.org/records/15309179. Source code for Slivka-bio can be found on the project repository: https://github.com/bartongroup/slivka-bio and https://zenodo.org/records/15304023. LIGYSIS-web is available at: https://www.compbio.dundee.ac.uk/ligysis/.
